# Editorial: Bio-based strategies for biotic and abiotic stress management in sustainable agriculture

**DOI:** 10.3389/fpls.2024.1536460

**Published:** 2025-01-15

**Authors:** Mustafa Yildiz, Gianfranco Romanazzi, Muhammet Cagri Oguz

**Affiliations:** ^1^ Faculty of Agriculture, Department of Field Crops, Ankara University, Dışkapı, Ankara, Türkiye; ^2^ Department of Agricultural, Food and Environmental Sciences, Marche Polytechnic University, Ancona, Italy

**Keywords:** bio-based strategies, biotic stress, abiotic stress, sustainable agriculture, plants

## Introduction

Bio-based applications and methods aimed at alleviating yield losses of plants under biotic and abiotic stress have important alternatives to chemical fertilizers. Developing bio-based strategies for the sustainability of agricultural production is of great importance for the environment, food and human health. In this context, microorganisms such as plant growth regulators bacteria (PGPB) and fungi have been studied intensively in recent years. In addition, biotechnological approaches such as bio-nanoparticles are being examined as sustainable and environmentally friendly methods in the interaction of water, soil and plants. Identifying and targeting the molecular, physiological, and biochemical changes caused by biotic and abiotic stresses in plants will effectively contribute to transforming bio-based approaches into products. The articles in this Research Topic contribute to the use of bio-based approaches in increasing tolerance to biotic and abiotic stress factors, reducing stress damage, protecting the environment and human health, and ensuring the sustainability of agricultural production.

Environmentally friendly and reproducible protocols are of great importance to ensure sustainable agricultural production under biotic and abiotic stress conditions. In this context, arbuscular mycorrhizal fungi (AMF) and plant growth promoting rhizobacteria (PGPR) can have plant-specific or broad-range effects. Raio et al. investigated the applicability of an epiphytic *Stenotrophomonas rhizophila* strain, which they isolated from the phyllosphere of an ornamental plant and named Ep2.2, as a plant protection agent. It was reported that *S. rhizophila* Ep2.2 was successful in inhibiting the *in vitro* growth of plant pathogenic fungi *Alternaria alternata* and *Botrytis cinerea* through the emission of volatile compounds.

In the other study, Peter et al. investigated the role of *Bacillus* sp. strain IPR-4 inoculated with melatonin (IPR-4/MET) in improving drought stress response in soybean. It was stated that the IPR-4/MET had significant effects on biochemical and physiological properties such as plant height, fresh weight, chlorophyll content, peroxidase, catalase, ascorbate peroxidase, and hydrogen peroxide. On the other hand, it was stated that stress-related molecular regulation was improved in IPR-4/MET treated plants. *Bacillus*–melatonin-treated plants recorded the highest expression levels (upregulated) of *GmCYP707A1* and *GmCYP707A2*, *GmPAL2.1*, and *GmERD1* in response to drought stress.

Collado-González et al. aimed to develop a production strategy to reduce nitrate pollution in production while transforming waste produced by industrial processing of celery into a value-added product. For this purpose, it was focused on developing sustainable agricultural production by reducing N used in agricultural areas using PGPB as a biostimulant. The study results reported that plants grown under limited N dose with applications showed higher antioxidant activity and total phenolic compounds content. The findings will contribute to the evaluation of celery by-products as an excellent source of bioactive compounds.

Nicotra et al. took a different approach to identify plant growth promoting rhizobacteria. It was reported plant growth was promoted by inoculating *Pseudomonas* and *Bacillus taxa*, and *Leclercia*, *Chryseobacterium*, *Glutamicibacter* and *Paenarthorbacter* genera into tomato rhizosphere. Furthermore, these PGPRs were reported to reduce the severity of *Fusarium* crown and root rot and bacterial spot infections. According to the characteristics determined by genome analysis, it is emphasized that the strains are functional in increasing/managing abiotic and biotic stress tolerance.

Studies on the use of chitosan in agricultural production, which has been the subject of many studies in terms of food and environmental safety in recent years, are important for sustainable agriculture. Rojas-Pirela et al. focused on the similarities between the effects of chitosan and PGPR on plant growth under biotic and abiotic stress conditions. In the study that gives a new direction to bio-based approaches; the emphasized similarities of PGPB and chitosan have important consequences for agricultural production. It is likely to make significant contributions to studies on the joint applicability of PGPB and chitosan.

In addition to abiotic stress factors such as drought, salt, and excessive heat, heavy metal toxicity causes yield losses in agricultural production. The fact that heavy metal accumulation poses a great threat to food safety and human health has focused studies on these stress factors. Anwar et al. reported that biochar, rhizobacteria and gibberellic acid applications increased tolerance to heavy metal stress [Cadmium (Cd), lead (Pb)] and drought stress in maize. The combined applications were reported to significantly improve shoot and root growth and biochemical parameters under stress conditions in addition to improved germination rates.

AMF are symbiotic soil fungi. Different plant species have different fungal species and genera in their roots. Addressing issues such as isolation, identification, usefulness and effects on stress tolerance of these fungi will contribute to sustainable agricultural production. Russo et al. attempted to determine the multitrophic interactions of the endophytic and entomopathogenic fungus *Beauveria bassiana* with tomato as a plant biostimulant and biological control agent. It was determined that *Beauveria bassiana* improved the physiological growth parameters of plants against *Botrytis cinerea*, which has pathogenic effects on tomatoes. It was also reported that Beauveria bassiana was successful against the biotic stress factor pathogen *B. cinerea*. Zhu et al. studied the effect of *Piriformospora indica* in alleviating salt stress. It was reported that *P. indica* significantly promoted soybean growth under mild saline-alkaline stress and showed significant positive effects on physiological and biochemical markers. *P. indica* is effective in increasing the adaptation ability of soybean to saline-alkaline stress by regulating ROS scavenging capacity, osmotic adjusting substance content and photosynthetic capacity.

The use of agricultural by-production and plant extracts to increase stress tolerance and reduce yield losses in agricultural production is among the important Research Topics. Gaucher et al. investigated the use of tomato and apple pomace in the development of a cutin-based biocontrol solution against an important apple disease caused by the biotic stress agent *Venturia inaequalis*. They reported that after different purification and formulation steps, the formulated cutin monomers could trigger a significant transcriptome reprogramming in apple plants and showed an antifungal effect on *V. inaequalis*.

Important approaches are being developed to evaluate biosynthesized nanoparticles in the protection of soil and water resources and as an alternative to chemical fertilizers in plants. Ishfaq et al. evaluating the wastewater treatment potential of MnO-NPs biosynthesized from *Bacillus* flexus strain, it was stated that MnO-NPs effectively reduced pollutants in wastewater. It was reported that a significant increase in physiological and antioxidant properties of wheat seedlings grown using treated wastewater was determined; oxidative stress values were reduced by 40%.

Commercial product development focuses on effective and sustainable bio-based solutions to ensure agricultural productivity and food security. Zuzunaga-Rosas et al. determining the effect of BALOX^®^, a plant-based biostimulant, on increasing tolerance to salt stress in lettuce seedlings; it was reported that the commercial biostimulant stimulated plant growth and the levels of Ca2+ and photosynthetic pigments. It was also stated that BALOX^®^ successful in reducing the accumulation of salt-induced stress biomarkers such as proline, malondialdehyde (MDA) and hydrogen peroxide (H_2_O_2_).

Identifying biotic and abiotic stress-related genes is important in testing new products and treatments to be developed to increase stress tolerance. Liu et al. conducted a study to determine the roles of metallothionein family genes in kiwi fruit in plant responses to cold stress (abiotic) and *Pseudomonas syringae* pv. *actinidiae* (Psa)-induced stresses (biotic). The genes identified in the study findings reported that they have protective roles in stress resistance provided through plant hormone-related signaling pathways.

Pierro et al. have reviewed effective and promising control strategies for the management of Bois noir (BN), which causes significant losses in fruit quality and yield in wine growing areas worldwide. In this article, innovative sustainable strategies developed to improve BN management, especially in the last two decades, are reviewed and discussed.

## Conclusion

Our knowledge of the role of PGPR and AMF in agriculture has increased significantly in the last decade. Besides, the development and application of new products such as bio-nanoparticles and bio-films revealed successful results. Research on the effects of bio-based strategies on stress tolerance will contribute to important results on the environment, food and human health. Undoubtedly, the information obtained from these studies will help in the application of beneficial microorganisms, products and methods in sustainable agriculture in the future.

